# The Future of Osteoarthritis Therapeutics: Emerging Biological Therapy

**DOI:** 10.1007/s11926-013-0385-4

**Published:** 2013-10-30

**Authors:** A. Mobasheri

**Affiliations:** 1 D-BOARD European Consortium for Biomarker Discovery, Faculty of Medicine and Health Sciences, The University of Nottingham, Nottingham, UK; 2 Arthritis Research UK Centre for Sport, Exercise and Osteoarthritis, The University of Nottingham, Nottingham, UK; 3 Arthritis Research UK Pain Centre, The University of Nottingham, Nottingham, UK; 4 Medical Research Council and Arthritis Research UK Centre for Musculoskeletal Ageing Research, The University of Nottingham, Nottingham, UK; 5 Medical Research Council and Arthritis Research UK Centre for Musculoskeletal Ageing Research, The University of Birmingham, Birmingham, UK; 6 Center of Excellence in Genomic Medicine Research (CEGMR), King Fahad Medical Research Center (KFMRC), King AbdulAziz University, Jeddah, 21589 Saudi Arabia; 7 Musculoskeletal Research Group, School of Veterinary Medicine and Science, Faculty of Medicine and Health Sciences, The University of Nottingham, Sutton Bonington Campus, Sutton Bonington, LE12 5RD UK; 8 Schools of Pharmacy and Life Sciences, University of Bradford, Richmond Road, Bradford, BD7 1DP UK

**Keywords:** Osteoarthritis, Therapeutics, Biological therapy, Therapeutic antibodies, Calcitonin, Kartogenin, Fibroblast growth factor 18 (FGF-18), Anticytokine therapy, Angiogenesis, Neurogenesis, DMOADs, DMARDs

## Abstract

Biological therapy is a thriving area of research and development, and is well established for chronic forms of rheumatoid arthritis (RA) and ankylosing spondylitis (AS). However, there is no clinically validated biological therapy for osteoarthritis (OA). Chronic forms of OA are increasingly viewed as an inflammatory disease. OA was largely regarded as a “wear and tear disease”. However, the disease is now believed to involve “low grade” inflammation and the growth of blood vessels and nerves from the subchondral bone into articular cartilage. This realization has focused research effort on the development and evaluation of biological therapy that targets proinflammatory mediators, angiogenic factors and cytokines in articular cartilage, subchondral bone and synovium in chronic forms of OA. This review article provides an overview of emerging biological therapy for OA, and discusses recent molecular targets implicated in angiogenesis and neurogenesis and progress with antibody-based therapy, calcitonin, and kartogenin, the small molecule stimulator of chondrogenesis.

## Introduction

The diagnosis, treatment, and rehabilitation of adults, adolescents and children with bone, joint or connective tissue disorders is a concern of clinicians and scientists working in rheumatology, traumatology, and orthopedics. The “musculoskeletal sciences” have become highly specialized areas of clinical medicine. For many years, most treatments of bone, joint, or connective tissue disorders have involved conventional pharmaceuticals, predominantly painkillers. OA therapy is a prime example. Acetaminophen relieves OA pain but does not reduce inflammation. It is effective for treating OA patients with mild to moderate pain. However, long-term acetaminophen use can cause liver damage. Nonsteroidal anti-inflammatory drugs (NSAIDs) reduce inflammation and relieve pain. Ibuprofen, naproxen, and stronger NSAIDs are effective for treating more chronic forms of OA pain. However, long-term consumption of NSAIDs can cause stomach upset, cardiovascular problems, gastric bleeding, and liver and kidney damage. Elderly people are particularly at risk of developing complications associated with NSAID use. Opioids and narcotics are used to treat more severe forms of OA pain. These strongest conventional drugs carry the serious risk of development of dependence, although this risk is believed to be relatively small for people with severe pain. Side effects of narcotics and opioids include nausea, constipation, and sleepiness.

The author has recently reviewed targeted pharmacological therapy for OA [1•]. However, currently available pain medications are not disease-modifying osteoarthritis drugs (DMOADs). The adverse side effects of these conventional drugs have shifted the focus of new therapeutics research to biological agents and therapy that uses biologicals or combinations of cells and biologicals. The transition from pharmacological to biological therapy will not be smooth. Also, biological therapy will not be suitable for all types of OA. Biological therapy is, effectively, a form of immunotherapy that has been used successfully for chronic forms of immune-mediated rheumatoid arthritis (RA), which is caused by excessive activity of the immune system. Although biological therapy may be a regarded as relatively new for treatment of musculoskeletal diseases, it has, in fact, been available for decades. Today’s biological therapy would not have been possible without the pioneering work of scientists including Edward Jenner and Paul Ehrlich. Biological therapy for RA includes etanercept, infliximab, adalimumab, and certolizumab, which target TNF-α, rituximab, which targets CD20-positive B cells, and tocilizumab, a humanized antibody against the IL-6 receptor. In RA treatment these drugs are taken in combination with methotrexate, a widely used disease-modifying anti-rheumatic drug (DMARD). However, because methotrexate is cytotoxic and has serious and potentially life-threatening side effects its use cannot be justified for OA. This paper will review recent developments and emerging concepts in biological therapy for OA.

## Calcitonin

As discussed in the first paper in this series [1•], recent studies have stressed the importance of the cartilage–bone interface in OA by demonstrating that cartilage and subchondral bone act as a single functional unit, in health and in disease. Subchondral bone has been identified as a priority target for new OA treatment [2]. Vascular pathology and the loss of mineral density in subchondral bone are important in the initiation and/or progression of OA [3]. Changes in subchondral bone may accelerate progression of pre-existing disease [4]. Therefore subchondral bone is an attractive target for developing DMOADs [2] and biological therapy.

Calcitonin is a 32-amino-acid polypeptide hormone produced in the parafollicular cells of the thyroid gland. It is a bone-density-conservation agent (http://www.ncbi.nlm.nih.gov/mesh/68050071) and has been shown to slow the bone-resorbing activity of osteoclasts while promoting the bone-building activity of osteoblasts. Therefore, calcitonin can cause marked transient inhibition of the ongoing bone resorptive process. It also helps to regulate blood calcium by reducing the amount of calcium released from the bones by working in the opposite way to parathyroid hormone (PTH) and 1,25-dihydroxyvitamin D. Calcitonin has been used clinically for treatment of hypercalcemia and osteoporosis and although it is predominantly used for postmenopausal osteoporosis, it can also be used for treating Paget’s disease, osteogenesis imperfecta, bone metastases, and malignancy-associated hypercalcemia.

Although calcitonin can be extracted from the ultimobranchial (thyroid-like) glands of salmon, for therapeutic purposes it is mainly produced by recombinant DNA technology or by chemical peptide synthesis, because the pharmacological properties of the synthetic and recombinant peptides are similar. Because calcitonin is a peptide, the most sensible method of administration is parenteral or intranasal. Miacalcin (calcitonin-salmon) produced by Novartis Pharmaceuticals is a nasal spray containing a synthetic polypeptide of 32 amino acids in the same linear sequence that is found in calcitonin of salmon origin. It is often prescribed for postmenopausal women who are at least five years past menopause and cannot, or do not wish to, take estrogen-containing products. Dosages for the nasal spray are typically 200 IU. Salmon calcitonin is also manufactured as a solution for injection under the skin (subcutaneously) or into the muscle (intramuscularly). However, these methods of administration hinder its clinical use. Adherence with therapy has been low, and withdrawal from clinical trials has been problematic [5]. Calcitonin has also been developed for oral consumption to improve patient acceptance and compliance, and it seems that oral formulations are rapidly absorbed with good bioavailability after consumption (reaching maximum concentration in 15 to 30 min) [6].

There are extensive published data on calcitonin from in-vitro and animal studies, and from clinical trials, on the effect of calcitonin on bone turnover, and compelling evidence to support its beneficial effects on bone mineral density and strength [7]. Evidence emerging from in-vitro, ex-vivo, and in-vivo studies and from preliminary clinical trials suggests that calcitonin treatment also has potential for the prevention and treatment of degenerative joint diseases, for example OA. A study published in 1999 attempted to relate calcitonin treatment to rate of bone resorption and serum levels of hyaluronan (HA) and antigenic keratan sulfate (KS) in an experimental model of canine OA [8]. Twenty-two dogs underwent anterior cruciate ligament transection (ACLT) and six underwent sham operation. Immunoassays were used to quantify hyaluronan (HA) and antigenic KS. All ACLT joints developed OA. In contrast with sham-operated animals, early and sustained increases in the levels of urinary and serum markers were observed for all the test dogs. Calcitonin therapy reduced the severity of OA changes in the cartilage lesions. Interestingly, longer durations of calcitonin therapy reduced the score for the OA lesions. The authors proposed that this form of therapy might have benefits for human subjects recovering from traumatic knee injuries.

In another clinical report Manicourt et al. [9] evaluated the effects of oral salmon calcitonin on Lequesne’s index scores and on biomarkers of joint metabolism in knee OA. The study was a randomized, double-blind trial of patients who received either placebo (*n* = 18), 0.5 mg sCT (*n* = 17), or 1 mg sCT (*n* = 18) daily for a period of 84 days. The biomarkers measured included C-telopeptide of type II collagen (CTX-II), type II collagen neoepitope C2C, matrix metalloproteinases MMP-1, MMP-3, MMP-8, and MMP-13, tissue inhibitors of metalloproteinases 1 and 2, and HA. By dissociating pain from the functional disability scores of the Lequesne’s index, the investigators revealed a significant improvement in the median functional disability score even after 42 days of treatment with calcitonin. Furthermore, significant biochemical responses were observed, including reduction in circulating MMP-13 and urinary excretion of CTX-II after 84 days of daily treatment with 1 mg calcitonin. The authors concluded, on the basis of the improved functional disability scores and reduced levels of catabolic biomarkers, that oral calcitonin might be useful for treatment of human knee OA [9].

A recent study suggests that an intra-articular salmon calcitonin-based nanocomplex reduces experimental arthritis [10]. Combinations of salmon calcitonin and hyaluronic acid (HA) attenuated joint inflammation in a mouse model of inflammatory arthritis [10].

Recent in-vitro studies have linked calcitonin with cartilage homeostasis and turnover [11]. Although the chondroprotective effects of calcitonin have not yet been demonstrated in humans, it is plausible that calcitonin may be important in cartilage biology and in treatment of OA [12]. The research team at Nordic Bioscience in Denmark has investigated the effects of salmon calcitonin on human cartilage explants [13]. Treatment with salmon calcitonin (100 pmol L^−1^–100 nmol L^−1^) increased proteoglycan and collagen synthesis in human OA cartilage as determined by measurement of proteoglycan synthesis by incorporation of radioactive labeled ^35^S labeled sulfate and ELISA quantification of collagen-type-II formation by pro-peptides of collagen type II (PIINP). These findings led the investigators to propose that salmon calcitonin may be beneficial for management of joint diseases by direct effects on chondrocytes [13]. Although it is debated as to whether the chondroprotective effect of calcitonin is mediated through subchondral-bone, directly on cartilage, or both in combination, it is clear that this hormone has direct effects on the cartilage component, although there is no evidence of direct effects on chondrocytes, because it has been reported that human cartilage and chondrocytes do not express the calcitonin receptor [14]. Calcitonin may exert a number of indirect effects through subchondral bone via induction of cAMP, resulting in attenuation of MMP-mediated cartilage degradation [10, 13, 15] or through calcitonin gene-related peptide (CGRP) receptors [16].

A recent clinical study of fifty Turkish female patients suggests that calcitonin inhalation therapy can relieve the pain associated with knee OA [17]. Although nasal calcitonin administered by inhalation at a dose of 200 IU did not alter serum IL-1β and MMP-3 levels, it did produce significant improvements in visual analogue scale (VAS), WOMAC pain, physical function scores, 20-m walking time, and WOMAC stiffness score. One of the weaknesses of this study was that the treatment group received nasal calcitonin by inhalation concomitantly with exercise therapy. It is, therefore, quite difficult to separate the effects of calcitonin inhalation therapy and physical exercise, although the placebo group also received exercise therapy. In addition to its effect on active osteoclasts, calcitonin has analgesic properties, possibly mediated through β-endorphins and the central modulation of pain perception [5]. Therefore, some its effects on OA pain may be mediated through β-endorphins and this should be the focus of future studies.

There have been few clinical trials of calcitonin with human subjects. A placebo-controlled 14-day clinical trial (clinicaltrials.gov identifier NCT00486369) was conducted by Nordic Bioscience to study the absorption, efficacy, and tolerance of oral calcitonin among patients with OA. The purpose of this clinical trial was to expose patients with OA to calcitonin and to determine plasma calcitonin levels after administration of 0.6 mg and 0.8 mg oral calcitonin. The study also assessed the effect of different doses of oral calcitonin (0.6 mg and 0.8 mg, oral) compared with placebo on serum CTX-I and CTX-II, and determined the tolerance profile of different doses and formulations of oral calcitonin compared with placebo [18]. The safety and efficacy of oral salmon calcitonin is being investigated in a two-year, multicenter, double-blind, placebo-controlled phase III clinical study of patients with knee OA [12]. Additional clinical trials with larger patient cohorts are needed to assess the clinical benefits of calcitonin treatment. Unfortunately, in two Phase III studies, oral calcitonin (0.8 mg with 200 mg 5-CNAC, once a day for postmenopausal OP and twice a day for OA) failed to meet key end points, and, in December 2011, Novartis Pharmaceuticals announced that it would not pursue further clinical development of oral calcitonin for postmenopausal OP or OA.

## Targeting Proinflammatory Cytokines and NF-κB

Strategies aimed at preventing excessive proinflammatory cytokine production, signaling, and downstream nuclear factor κB (NF-κB) activation, by use of highly specific drugs, small interfering RNAs (siRNAs), or other biological inhibitors [19], are the focus of current OA research. Some of these biological inhibitors may come from natural products, plants, or herbs [20, 21]. Because these cannot be strictly classified as “biological therapy”, they will not be covered in this review. Biological therapy capable of blocking cytokine action and NF-κB signaling may be a promising means of treatment of OA.

## New Antibody-Based Therapy

Antibody therapy for treatment of chronic forms of OA is becoming a reality. Recent studies have demonstrated that OA has a significant inflammatory component [22•]. OA is associated with increased expression and activity of several secreted proinflammatory cytokines in joint tissues. These cytokines activate catabolic pathways and promote the production of matrix-degrading enzymes. In RA, interleukin 1β (IL-1β) and tumor necrosis factor α (TNF-α), crucial cytokines involved in degeneration of the articular cartilage matrix, are required for full expression of rheumatoid disease [23]. There is increasing evidence in support of the idea that proinflammatory cytokines are important not only in inflammatory arthritis but also in degenerative joint diseases [24–26]. Molecular analysis of cytokine mRNA and protein expression in RA tissue has revealed that other proinflammatory cytokines, including IL-6, GM-CSF, and chemokines such as IL-8, are also abundant in patients [27]. These molecules and chemokines are increasingly being identified in studies of joint tissues from OA patients [28]. These studies suggest that biological therapy should target the proinflammatory cytokines and chemokines involved in promoting the progression of joint disease.

The realization that the removal or neutralization of TNF-α from the diseased host prevents development of the illness [29] has been important for integration of biological therapy in rheumatology, subsequent progress, and development of antibody-based therapy. Consequently, this cytokine and its receptor have been the focus of intensive research, especially in the context of rheumatic and autoimmune diseases [30]. The rationale for targeting TNF-α was initially provided by in-vitro studies which demonstrated that anti-TNF-α antibodies added to cultures of cells derived from diseased joints can inhibit the spontaneous production of pro-inflammatory cytokines [31]. This makes them and their downstream signaling pathways prime targets for novel therapeutic strategies [32]. Apart from IL-1β and TNF-α, several other cytokines and chemokines, including IL-6, IL-8, and IL-17, are implicated in OA. These proinflammatory cytokines bind to their respective cell-surface receptors and activate inflammatory signaling pathways culminating in activation of NF-κB a transcription factor that can be induced by stress-related stimuli, including excessive mechanical stress and extracellular matrix (ECM) degradation products. Once activated, NF-κB regulates the expression of many cytokines, chemokines, adhesion molecules, inflammatory mediators, and several matrix-degrading enzymes. Therefore, proinflammatory cytokines, their cell-surface receptors, and NF-κB and associated signaling pathways are obvious therapeutic targets in OA.

Published case studies report successful treatment of debilitating pain resulting from severe OA by use of monoclonal antibodies to cytokines [33]. Published preclinical studies also suggest that monoclonal antibodies and single-chain Fv antibody (scFv) against TNF-α can potently inhibit inflammation and prevent cartilage damage initiated by this cytokine [34]. In contrast with full-length IgG, ESBA105 also penetrates into cartilage and can be expected to reverse the TNF-α-induced catabolic state of articular cartilage in arthritic diseases. These studies recognized the value of anti-TNF-α therapy as a treatment option for severe OA and proposed that larger controlled trials should be established to investigate this possibility. Clearly, this approach should be selectively applied to severe OA cases where there is a strong inflammatory component.

Infliximab and etanercept are anti-TNF-α therapy approved by regulatory authorities in the US and Europe for treatment of RA [31]. Therefore, anti-cytokine therapy is a significant new addition to available therapeutic options for RA [31]. Randomized phase II and III clinical trials of infliximab and etanercept have demonstrated an acceptable safety profile and marked clinical efficacy, especially in cases that have not responded adequately to conventional therapy with methotrexate [32].

In OA, proinflammatory cytokines are the crucial biochemical signals that stimulate chondrocytes to release cartilage-degrading proteinases [24–26]. The rationale for use of anticytokine therapy in OA is based on extensive evidence from in-vitro and in-vivo studies that demonstrated specific effects of the proinflammatory cytokines IL-1β and TNF-α in the initiation and progression of articular cartilage destruction [25, 35]. Further evidence suggests that, in addition to IL-1β and TNF-α, other pro-inflammatory cytokines, including IL-6, members of the IL-6 protein superfamily, IL-7, IL-17, and IL-18, are also capable of promoting cartilage degradation [35]. These cytokines may synergize with IL-1β and TNF-α to amplify and accelerate cartilage destruction [35]. Other cytokines released during the inflammatory process in the OA joint may be regulatory (IL-6, IL-8) or inhibitory (IL-4, IL-10, IL-13, and interferon-γ (IFN-γ)) [24]. Goldring (2001) has suggested that therapeutic intervention with the purpose of blocking or reversing structural damage is likely to be more effective when there is a possibility of preserving normal homeostasis [25], enabling anabolic activity to effectively “catch-up” with catabolic reactions in the joint. This approach would attempt to restore physiological functions in the joint and to block catabolic pathways activated by inflammatory mediators. Therefore cytokine targeting must be specific, avoiding the inhibition of anti-inflammatory cytokines that may be involved in repair responses as endogenous therapeutic agents for counteracting cartilage destruction in OA [24, 25]. It is important to stress that OA is a disease of the whole joint, including cartilage and synovium [36•]. The inflammatory role of the synovium in OA is becoming more established in the field of rheumatology [22•, 37]. Synovitis involves engagement of Toll-like receptors and activation of the complement cascade by degradation products of the extracellular matrix of cartilage and other joint structures [38]. The ensuing synovial reaction leads to the synthesis and release of a variety of cytokines and chemokines [22•, 38, 39]. These catabolic and inflammatory mediators are all potential targets for therapeutic intervention [38].

Therapeutic strategies that concurrently use growth factors, for example transforming growth factor-beta (TGF-β), insulin-like growth factor-1 (IGF-1), fibroblast growth factor-2 (FGF-2), platelet-derived growth factor (PDGF), and connective tissue growth factor (CTGF), may be required in advanced cases of OA in which the repair responses of the cartilage may be severely compromised [35]. The heparin-binding fibroblast growth factor family of proteins are hormone-like modulators of cell proliferation and differentiation in vitro and in vivo [40]. These growth factors and their relatively high-affinity cell-surface receptors are essential for mammalian development [41]. Three members of the fibroblast growth factor (FGF) family, FGF-2, FGF-18, and FGF-8, have been implicated in cartilage homeostasis [42]. Fibroblast growth factor-18 (FGF-18) is a trophic factor for mature chondrocytes and their progenitors, and stimulates chondrogenesis and cartilage repair in animals model of injury-induced OA [43, 44]. FGF-18 may have a dual function in OA, because it has the capacity to promote the formation of new bone, including bony spurs and subchondral sclerosis [45] rather like hedgehog, which can lead to either catabolic or anabolic joint remodeling, depending on the presence of other factors. Enzymatic disruption, degradation, or removal of these growth factors, or disruption of their function, as in the enhanced binding of free IGF-1 with IGF binding proteins in OA joint synovial fluid, may compromise and ultimately be responsible for the inadequate repair of articular cartilage in OA [35].

## Tanezumab for the Treatment of Painful Knee OA

A recent proof-of-concept clinical trial investigated the safety and analgesic efficacy of treatment with tanezumab, a humanized monoclonal antibody that binds to and inhibits nerve growth factor (NGF). The results of the trial, which was led by Dr Nancy Lane, were published in the New England Journal of Medicine in 2010 [46••]. The authors report that blocking the pain-related activity of NGF with the neutralizing humanized monoclonal antibody tanezumab can relieve knee OA pain. The investigators randomly assigned 450 patients with knee OA to receive tanezumab (administered at doses of 10, 25, 50, 100, or 200 μg per kilogram of body weight) or placebo on days 1 and 56. The primary efficacy measures were knee pain while walking and the patient’s global assessment of response to therapy. The investigators also assessed pain, stiffness, and physical function by use of WOMAC; response using the criteria of the Outcome Measures for Rheumatology Committee and Osteoarthritis Research Society International Standing Committee for Clinical Trials Response Criteria Initiative (OMERACT-OARSI); and safety. Tanezumab, as compared with placebo, was associated with a reduction in joint pain and improvement in function, with mild and moderate adverse events, among patients with moderate-to-severe knee OA. This study raised the exciting possibility of using neutralizing antibodies therapeutically, similar to the use of antibodies against TNF for patients with RA [47]. Although tanezumab was highly effective in treatment of pain and functional impairment of patients with hip and knee OA, clinical trials of the drug were halted by the US Food and Drug Administration (FDA) in 2010 after 87 cases of osteonecrosis were reported in nearly 7,000 patients treated with different doses of the drug. An independent adjudication committee (IAC) has since determined that only 2 of those 87 cases were treatment-induced osteonecrosis. However, it was concluded that tanezumab causes rapid worsening of OA in 68 patients treated at the highest doses and in combination with NSAIDs.

## Targeting Angiogenesis and Neurogenesis

It has been proposed that the growth of blood vessels (angiogenesis) and nerves (neurogenesis) from the subchondral bone into articular cartilage may mediate the association between joint pathology and pain symptoms in OA [48]. In OA, angiogenesis is increased in the synovium, osteophytes and menisci and may lead to ossification in osteophytes and in the deep layers of articular cartilage. This is another example in which subchondral bone comes into sharp focus, emphasizing the importance of the osteochondral interface in OA [49, 50]. Studies of angiogenesis in rodent models of OA suggest that changes in vascularization occur early during the development of OA, especially in the rat [51]. Although both angiogenic and antiangiogenic factors are upregulated in OA joints, vascular growth seems to predominate, and the articular cartilage loses its resistance to vascularization [52]. Expression of NGF and the sensory nerve growth it stimulates are believed to link osteochondral angiogenesis to pain in different forms of arthritis including OA [48]. In addition, inflammation drives synovial angiogenesis by activation of macrophages [52]. Angiogenesis and nerve growth are linked by common pathways that involve the release of proangiogenic factors, for example vascular endothelial growth factor (VEGF), NGF, and neuropeptides including substance P, corticotropin-releasing factor, urocortin, and vasoactive intestinal peptide [37, 52].

Studies of humans have shown that increased vascular penetration and nerve growth expression in the meniscus is a potential source of pain in knee OA [53]. Angiogenesis and associated sensory nerve growth in menisci may not only contribute to pain but also to further inflammation and tissue damage [54], particularly at the osteochondral junction, driving disease progression in knee OA [48, 49, 52, 53]. Similar inflammatory mechanisms may stimulate angiogenesis in the synovium [55] contributing to joint effusion through impaired synovial fluid drainage [56].

In summary, innervation accompanies vascularization and inflammation. Targeting and inhibiting angiogenesis may therefore help identify new therapeutic strategies for treating OA [57]. Blocking angiogenesis by use of novel antiangiogenic therapy and inhibiting or neutralizing proangiogenic and neurogenic factors may therefore reduce the burden of inflammatory joint disease in RA and OA. Earlier this year the FDA Arthritis Advisory Committee met to discuss the anti-NGF class of drugs currently under development, and associated safety issues. The panel concluded that the potential benefits of experimental anti-NGF drugs clearly outweigh the risks associated with the treatments [58]. These developments are likely to have a significant effect on future research and development at Regeneron Pharmaceuticals, Pfizer, and Johnson and Johnson, working on pain management for patients with back pain and OA. Future therapeutics that target nerve growth will benefit from the recent FDA decision on the future development of NGF blockers. Therefore, future clinical trials are likely to focus on anti-NGF therapy for both RA and OA.

Inhibition of NGF and NGF-stimulated nociceptive pathways in OA appears to be effective. However, the adverse effects of NGF blockage require further investigation [59].

## Conclusions

**Fig. 1:**
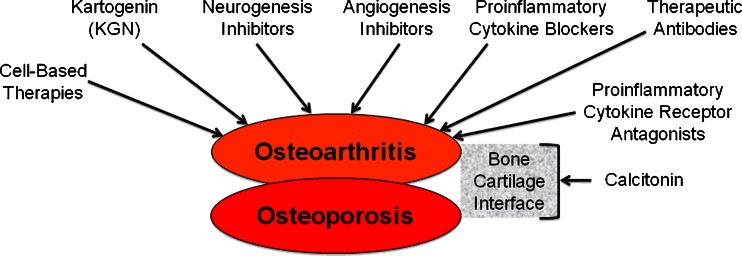
Schematic diagram summarizing current concepts in the biological treatment of OA

A large and unmet need exists for therapeutic intervention for OA [60]. Biological therapy has revolutionized the treatment of RA in the last decade. The concept of applying biological therapy to OA is not new. The schematic diagram shown in Fig. 1 summarizes current concepts in the biological treatment of OA.

Developing anticytokine therapy for OA was proposed several years ago [25]. However, the transfer of information from RA therapy to OA therapy has been slow. As with conventional drugs, a variety of important safety concerns will affect the choice and use of biological agents. The most significant of these include increased the risk of infection and malignancy and adverse reactions to the initial administration [61]. Biological therapy could potentially be used for chronic forms of OA among patients that may have previously had RA (especially patients who have not responded to other forms of therapy). Some of these patients may have previously had early RA that was treated and resolved before developing into chronic RA. OA involves three main tissues in the synovial joint: articular cartilage, bone, and synovium [62]. Biological therapy may have benefits for some or all of these tissues. The presence of “systemic inflammation” in OA of some patients may provide a rationale for biological therapy. It is important to clarify that it is virtually impossible to reverse cartilage damage at late and chronic stages of the disease. Also, biological therapy is probably not going to be suitable for less severe forms of OA, which can be treated with conventional and complementary treatment. Therefore, understanding the risks and benefits of using biological therapy for OA will be a important priority of future studies.

Future research must be directed toward defining the risk-to-benefit ratio for biological therapy, especially if the purpose of the therapy is to target mediators of “low grade” inflammation, especially for obese patients with insulin resistance and diabetes [63]. This will be extremely challenging, because mediators of “low grade” inflammation are likely to have important physiological effects on other organ systems. Anti-NGF drugs [58] and angiogenesis inhibitors (http://www.cancer.gov/cancertopics/factsheet/Therapy/angiogenesis-inhibitors) [64] are being developed by cancer researchers and are primarily intended for treatment of neoplastic diseases, but some of these agents may also find applications in other areas of medicine including rheumatology.

**Fig. 2:**
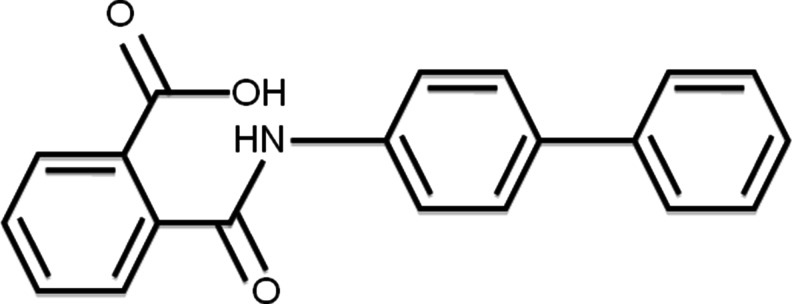
Molecular structure of kartogenin ((2-[(biphenyl-4-yl)carbamoyl]benzoic acid; 4′-phenylphthalanilic acid (8CI); also known as KGN). Kartogenin is a cell-permeable biphenylcarbamoylbenzoate compound that potently induces the differentiation of mesenchymal stem cells (MSCs) into chondrocytes (EC50 = 100 nmol L^−1^). It binds reversibly to the FC-1 fragment of filamin A and disrupts its association with core-binding factor β subunit (CBFβ) leading to the nuclear localization of CBFβ and binding to runt-related transcription factor (RUNX) to regulate chondrogenesis. PubChem CID: 2826191

The next review in this series of three articles will deal with preventive strategies and cell-based therapy for OA. Cell-based therapy using chondrocytes and stem cells are effectively another form of “biological therapy”. This is an exciting but highly controversial area. One of the most interesting areas of research is the work that has been conducted with the small molecule kartogenin (Fig. 2).

Johnson and colleagues identified kartogenin by image-based high-throughput screening, and found it has chondroprotective effects in vitro, and is efficacious in OA animal models of OA [65••]. Kartogenin can therefore replenish cartilage from endogenous stem cells by inducing the selective differentiation of multipotent mesenchymal stem cells (MSCs) into chondrocytes [66]. Information is available about the chondrogenic mode of action of kartogenin. It binds filamin A, disrupts its interaction with the transcription factor core-binding factor β subunit (CBFβ), and induces chondrogenesis by regulating the CBFβ and runt-related transcription factor-1 (RUNX1) transcriptional program [65••]. This recent work has generated much excitement about the potential for harnessing the potential of stem cells for cartilage repair [67]. This work invigorates research into small-molecule therapy and regenerative medicine for OA [68]. It also provides new insights into the control of chondrogenesis that may ultimately lead to a stem cell-based therapy for OA. Kartogenin and other structurally related small molecules that can promote selective differentiation of MSCs into chondrocytes may prove to be extremely useful for improving the outcome of cell-based therapy by stimulating endogenous mechanisms for repair of damaged cartilage, thus enhancing the joint’s intrinsic capacity for cartilage repair.
